# TGF-Β1 & PNPLA3 Genetic Variants and the Risk of Hepatic Fibrosis and HCC in Egyptian Patients with HCV-Related Liver Cirrhosis

**DOI:** 10.31557/APJCP.2021.22.10.3317

**Published:** 2021-10

**Authors:** Azhar Mohamed Nomair, Lamia Said Kandil, Hanan Mohamed Nomeir, Noha Said Kandil

**Affiliations:** 1 *Department of Chemical Pathology, Medical Research Institute, Alexandria University, Egypt. *; 2 *Department of Pharmacology and Therapeutics, Faculty of Pharmacy, Pharos University, Egypt. Lecturer in the School of Biological Sciences, Faculty of Science, University of East Anglia, UK. *; 3 *Department of Medical Biochemistry, Faculty of Medicine, Alexandria University, Egypt. *

**Keywords:** Gene polymorphism, liver fibrosis, HCC, HCV

## Abstract

**Objective:**

The clinical outcomes of hepatitis C virus (HCV) infection and its sequelae including liver cirrhosis and hepatocellular carcinoma (HCC) are greatly affected by host genetic factors; however, the possible mechanisms are still largely unclear. This work aimed to assess transforming growth factor-β1 (TGF-β1), and patatin-like phospholipase domain containing-protein 3 (PNPLA3) genetic variants as risk factors for hepatic fibrosis and hepatocellular carcinoma (HCC) in Egyptian patients with HCV-related liver cirrhosis.

**Methods:**

Seventy HCV-related liver cirrhosis patients (Total cirrhosis) who were divided into two groups; 34 patients with HCC (HCC group), and 36 patients without HCC (LC group) and 20 healthy volunteers (control group) were included. Routine laboratory investigations and imaging studies were determined. TGF-β1 (Arg25Pro; 915G>C) and PNPLA3 (I148M; C>G) variants were evaluated using real-time polymerase chain reaction (real-time PCR).

**Results:**

HCC group showed a significantly higher GG genotype distribution of TGF-β1 (Arg25Pro) than the LC group (P= 0.008, OR: 7.083, CI 95%: 1.422 – 35.282). The distributions of GG genotype (P= 0.047) and G allele (P= 0.002, OR: 4.395, CI 95%: 1.622 – 11.911) of PNPLA3 (I148M) were significantly higher in total cirrhosis patients than controls.

**Conclusion:**

TGF-β1 (Arg25Pro) GG variant may be associated with HCC risk in HCV-related liver cirrhosis patients, while PNPLA3 (I148M) GG variant may be associated with cirrhosis development but not HCC risk in HCV-related liver cirrhosis patients.

## Introduction

It was estimated that Hepatitis C virus (HCV) affects about 175 million people all over the world. 80% of these individuals developed chronic HCV infection, which is considered one of the major causes of liver fibrosis, deformation of hepatic structure, and consequently progression to nodular formation and hepatic cirrhosis (Mohy and Fouad, 2014). Moreover, chronic necro-inflammation or constant cell death, compensatory hepatic regeneration and activation of non-parenchymal cells, together with an altered immune response, aggravate liver fibrosis and promote tumorigenesis (Ringelhan et al., 2018). Thus Hepatitis C virus infection is assumed to be the world’s main cause of hepatocellular carcinoma (HCC) worldwide. However, the genetic predisposition and understanding of the cellular and the molecular mechanisms involved in hepatic fibrogenesis, and HCC development need to be further investigated (Tan et al., 2019). 

Hepatocytes bear various cytokine receptors. Moreover, the sinusoidal endothelial cells of the liver are considered producers and targets for different cytokines. Therefore, cytokines have been involved in hepatocyte development and regeneration. They are released in response to a diverse range of cellular stresses including infection, inflammation, and carcinogen-induced injury, and they can modulate the triggered immune response. Hence, polymorphisms in cytokine genes or variations in their expression may contribute to the pathogenesis of liver-related diseases such as fibrosis and cirrhosis, and promote carcinogenesis through the induction of chronic inflammatory states (Budhu and Wang, 2006)

Transforming growth factor-beta 1 (TGF-β1) which is one of three isoforms of TGF-β family is a multi-functional cytokine. It is a main regulator of cell growth, differentiation, angiogenesis, extracellular matrix formation, immune response regulation, and cancer development. It is a central regulator in chronic liver diseases and its role in modulating liver fibrosis progression has been investigated (Mohy and Fouad, 2014). The changes in its secretion may cause dysregulation of the host immune response in chronic HCV patients (Kondo et al., 2011). It was also found that TGF-β1 expression was up regulated in many tumors including HCC (Guo et al., 2018)

TGF-β1gene is encoded on chromosome 19q13, and several genetic variants in the TGF-β1 gene have been identified. The Arg25Pro (915G>C) variant in TGF-β1exon 1 is present in the signal peptide sequence that is separated from the TGF-β1 precursor at codon 29 level. The Arg25Pro variant corresponds to a replacement of the large polar amino acid arginine by the small non-polar proline near the 3’ end of the hydrophobic center of the signal sequence (Stoll et al., 2004). It was established that a homozygous (Arg25Pro) GG genotype is associated with higher TGF-β1 levels than a heterozygous (Arg25Pro) GC genotype. Therefore, the correlation between that genetic variant and disease status has been studied in a various range of diseases, and its effect on hepatic fibrosis progression and HCC risk revealed controversial results (Tag et al., 2003).

Patatinlike phospholipase 3 (PNPLA3), alternatively referred to as adiponutrin, encodes a 481amino acid protein of unknown function that belongs to the PNPLA3 domaincontaining family. PNPLA3 is a predominantly liverexpressed transmembrane protein that plays a vital role in the lipid synthesis by converting lysophosphatidic acid into phosphatidic acid. It is located on the chromosome 22 (22q13.31) containing 9 exons (Medrano et al., 2017). 

The cytosine to guanine substitution results in an isoleucine switch at codon 128. Recently, the rs738409 C > G polymorphism in the PNPLA3 gene also referred as (I148M) variant has been correlated to increasing the risk for HCC development and high risk of hepatic steatosis, portal inflammation, and fibrosis. It has been discovered that this variant is related to triglycerides accumulation in the hepatocytes as it decreases its hydrolysis level, and it confers increased risk of liver disease progression. The PNPLA3 (I148M) variant has also been linked to fibrosis severity and HCC risk in patients with known liver disease (He et a., 2010; Singal et al., 2014). However, controversial conclusions were given especially for the association of this polymorphism with hepatitis C virus (HCV)-related HCC because of limited sample sizes and ethnic variation.

The aim of the present work is to study the relation between the genetic variant in codon 25 of TGF-β1 gene (Arg25Pro; 915G>C), and the PNPLA3 (I148M; C>G) variant in the PNPLA3 gene and liver fibrosis development, HCC risk, and the severity of liver disease among Egyptian patients with HCV-related liver cirrhosis.

## Materials and Methods

*Subjects*

After the approval of the Ethical Committee of the Medical Research Institute, ninety subjects were included in this study. It was conducted in accordance with the guidelines of the Helsinki Declaration of the World Medical Association’s “Ethical Principles for Medical Research Involving Human Subjects”. All patients were recruited from Hepatology unit of Medical Research Institute, Alexandria University. Written informed consents were taken from all subjects before participation in the study. 

The subjects of the study were divided into two groups. A group of seventy patients with HCV-related liver cirrhosis (total cirrhosis) who were subdivided into 34 patients with HCC (HCC group) and 36 patients without HCC (LC group), and a group of twenty apparently healthy volunteers with no history of liver diseases, free on clinical examination and with normal liver function tests as a control group. All subjects were of comparable age and socioeconomic status. The diagnosis of liver cirrhosis was based on clinical, laboratory and ultrasound findings. HCC patients were diagnosed by detecting a hepatic focal lesion by ultrasound, and confirmed by a contrast-enhanced triphasic computed tomography (CT) scan study or magnetic resonance imaging, and this is in accordance with the American Association for the Study of Liver disease (AASLD) guidelines (Bruix et al., 2011). Staging of HCC cases was based on The Barcelona Clinic Liver Cancer (BCLC) system (Forner et al., 2010). 

Exclusion criteria included patients with Hepatitis B viral infection, or other forms of chronic liver diseases including autoimmune hepatitis, and drug-induced liver disease, and patients had evidence of alcohol intake. 

**Materials and Methods**

To all the studied subjects, the following was done: Full history taking, complete physical examination, and abdominal ultrasound. Liver echo pattern was recorded to confirm the presence or absence of cirrhosis and / or periportal fibrosis. It was also used to detect portal hypertension, portal vein thrombosis, ascites, splenomegaly and focal lesions. Child-Turcotte-Pugh (CTP) score and class were calculated for all cases enrolled in the study to estimate the severity of liver injury (Cholongitas et al., 2005).

*Biochemical Analyses*

Fasting blood samples were collected from all subjects to perform routine laboratory investigations in the form of complete blood count (CBC), aspartate and alanine aminotransferases (AST, ALT), alkaline phosphatase (ALP), total and direct bilirubin (T-Bil, D-Bil), gamma glutamyl transferase (GGT), total cholesterol (TC), triglycrides (TG), prothrombin time (PT), international normalized ratio (INR) and alfa fetoprotein (AFP). 

For estimation of the degree of fibrosis we used AST to platelet ratio index (APRI), which was calculated as follows: APRI= [(AST/upper limit of normal (ULN) (U/L)/platelet count (109/L)] × 100 (ULN of AST was 35 U/L for male patients, and 31 U/L for female patients.). The staging of fibrosis was as follows: ≤0.5= F0, >0.5 – ≤1= F1, >1 – ≤1.5= F2, >1.5 – ≤2= F3, >2= F4 (Shah et al., 2009).

*Molecular genetic studies*

Molecular genetic studies were performed as follows; DNA samples were isolated from the whole blood by using DNA extraction kit; the Gene JET_Genomic DNA Purification Kit (Thermo Fisher Scientific), according to the manufacturer’s instructions. The concentration and purity of the extracted genomic DNA was determined using the Thermo Scientific NanoDrop™ 1000A Spectrophotometer at 260 and 280 nm.

Single nucleotide gene polymorphism (SNP) genotyping analysis was performed on each DNA sample using TaqMan allelic discrimination real-time polymerase chain reaction (real-time PCR) and dual labeled fluorogenic TaqMan probes SNP genotyping technology on a StepOne Real-Time PCR System (Applied Biosystems, USA).

A predesigned and validated TaqMan SNP Genotyping Assays (C_11464118_30; catalog number: 4351379; Thermo Fisher Scientific) for TGF-β1 (Arg25Pro) variant, and (C_7241_10; catalog number: 4351379; Thermo Fisher Scientific) for PNPLA3 (I148M) variant, was used and conducted according to the manufacturer’s protocols. The sequence used for the assay design of TGF-β1 (Arg25Pro) variant was: CAGGTGGATAGTCCCGCGGCCGGC[G/C]GGCCAGGCGTCAGCACCAGTAGCCA, and for PNPLA3 (I148M) variant was: AGGCCTTGGTATGTTCCTGCTTCAT[C/G]CCCTTCTACAGTGGCCTTATCCCTC. The assays were performed for each sample as well as for a no template control. 

The reaction mixture of a net volume of 20 μL for each assay consisted of 10 μL of TaqMan Genotyping Master Mix (catalog number: 4371353; Thermo Fisher Scientific), 3.0 μL of the extracted DNA, 1.0 μL of 20× SNP assay mixture (counting the two primers and probes) and 6.0 μL of nuclease-free water. Thermal cycling conditions were advanced as follows: 10 minutes at 95 °C for enzyme activation, followed by 40 cycles of 15 seconds at 95°C, and one minute at 60°C for annealing.

Data analysis was performed for genotyping using TaqMan Genotyper Software (Applied Biosystems), and allelic discrimination plots were generated for each run to visualize the genotypes of the entire cohort.

*Statistical analysis*

The data was analyzed using version 20.0.0 of the IBM SPSS software package. (NY: IBM Corp, Armonk). The Kolmogorov-Smirnov test was used to check the normality of distribution of variables; the Chi-square test was used to determine comparisons between classes of categorical variables. Hardy-Weinberg equilibrium was used to compare the observed genotype frequencies with the predicted ones among the control subjects. Genotypic associations of the genetic variants were evaluated using Chi-square test, followed by risk assessment using odds ratio and 95% confidence interval (CI). For normally distributed quantitative variables, student t-test was used to compare two groups, while Mann Whitney test was used to compare two groups for non-normally distributed quantitative variables. ANOVA was used to compare the three studied groups and followed by Post Hoc test (Tukey) for pairwise comparison. Different groups for abnormally distributed quantitative variables were compared by Kruskal Wallis test followed by Post Hoc test (Dunn’s for multiple comparisons test) for pairwise comparison. Significance of the obtained results was judged at the 5% level.

## Results

Total seventy patients with HCV-related liver cirrhosis and 20 healthy volunteers were enrolled in this work. The patients were subdivided into two groups; HCC group, their mean age was 61.2 ± 5.4 years, and LC group, their mean age was 57.9 ± 7.7 years. The mean age of the control group was 57.2 ± 6.5 years. 64.7% of HCC group was males, while 70% of the control group was females. There was significant difference between HCC and LC groups regarding ALT, AST, T-Bil, D-Bil, GGT, and WBC count. The demographic and laboratory data of the studied groups are summarized in Table 1. 

The clinical and radiological characteristics of the studied groups are presented in Tables 2 and 3. There was non-significant difference between HCC and LC groups regarding child class (P=0.655), child score (P= 0.271), or ascites grade (P= 0.321). However, there was significant difference between the two groups regarding the presence of portal vein thrombosis and splenomegaly (P= 0.002, P= 0.002 respectively).

BCLC staging of HCC patients revealed that 70.6% were in the advanced and end-stage of the disease, while 29.4% were in early and intermediate stages. Triphasic CT evaluation of HCC patients showed that the majority of them (35.3%) had more than 3 focal lesions on presentation. lymph node involvement was in 23.5%, and extrahepatic spread was in 5.9% of HCC patients (Table 3).

The results of genotyping and allele frequency analysis of TGF-β1 (Arg25Pro) and PNPLA3 (I148M) variants are displayed in Tables 4 and 5.

Regarding genotyping distribution and allele frequency of TGF-β1 (Arg25Pro) variant, non-significant difference was found between total cirrhosis patients and controls (Table 4). However, GG genotype distribution of TGF-β1 (Arg25Pro) was significantly higher in HCC group than LC group (OR: 7.083, CI 95%: 1.422 – 35.282) (Table 5). Moreover, it was significantly associated with low albumin levels (P= 0.001), while there was no association between GG genotype of TGF-β1 (Arg25Pro) and APRI score (P= 0.240) (Table 6).

The distribution of GG genotype and G allele of PNPLA3 (I148M) were significantly higher in total cirrhosis patients than controls (P= 0.047), (OR: 4.395, CI 95%: 1.622–11.911) respectively (Table 4). However, there was non-significant difference in genotyping or allele frequency of PNPLA3 (I148M) variant when comparing HCC group with LC group (Table 5). Also, PNPLA3 (I148M) variant was not associated with Child class (P= 0.894), Child score (P= 0.993), or APRI score (P= 0.165) (Table 6). 

**Table 1:** Demographic and Laboratory Data of the Studied Groups

	HCC group (n = 34)	LC group (n = 36)	Total cirrhosis patients (n = 70)	Control group (n = 20)	*P1*	*P2*	*P3*	*P4*
Gender, n (%)								
Male	22 (64.7%)	18 (50%)	40 (57.1%)	6 (30%)	0.214	0.014*	0.147	0.032*
Female	12 (35.3%)	18 (50%)	30 (42.9%)	14 (70%)				
Age^b^	61.2 ± 5.4	57.9 ± 7.7	59.5 ± 6.9	57.2 ± 6.5	0.0501			0.177
ALT (U/L)^a^	51(23 – 291)	34.5(12 – 234)	35(12 – 291)	18(12 – 20)	0.002*	<0.001*	0.001*	<0.001*
AST (U/L)^a^	85(32 – 381)	53(21 – 312)	67(21 – 381)	15(11 – 22)	0.013*	0.001*	<0.001*	<0.001*
Albumin (g/dl)^b^	2.2 ± 0.42	2.3 ± 0.37	2.3 ± 0.40	4.4 ± 0.17	0.401	<0.001*	<0.001*	<0.001*
T-Bil (mg/dl)^a^	5.6(0.80 – 19.1)	2.3(0.50 – 28.1)	2.7(0.50 – 28.1)	0.65(0.50 – 0.90)	0.013*	0.001*	<0.001*	<0.001*
D-Bil (mg/dl)^a^	2.9(0.10 – 12.7)	1.2(0.20 – 22.6)	1.7(0.10 – 22.6)	0.10(0.10 – 0.20)	0.037*	0.001*	<0.001*	<0.001*
ALP (U/L)^a^	112(46 – 275)	89(33 – 256)	98(33 – 275)	99.5(88 – 120)	0.202			0.854
GGT (U/L)^a^	56(20 – 164)	36(14 – 176)	45(14 – 176)	19(10 – 22)	0.047*	0.001*	0.001*	<0.001*
TC (mg/dl)^b^	157.6 ± 60.6	159.2 ± 50.8	158.4 ± 55.4	165.7 ± 19.3	0.844			0.364
TG (mg/dl)^b^	126.6 ± 45.5	120.4 ± 29.1	123.4 ± 37.8	118.5 ± 21	0.651			0.577
INR^b^	1.6 ± 0.25	1.6 ± 0.46	1.6 ± 0.38	0.97 ± 0.01	0.75	<0.001*	<0.001*	<0.001*
Hb (g/dl)^b^	10.5 ± 1.5	10 ± 1.5	10.3 ± 1.5	13.4 ± 1.03	0.248	<0.001*	<0.001*	<0.001*
WBC count (x10^3^/μL)^a^	8.9(2.9 – 30.4)	8(2.6 – 16.4)	8.4(2.6 – 30.4)	6.2(4.9 – 9.5)	0.038*	0.001*	0.1	0.006*
Platelets count (x10^3^/μL)^b^	123 ± 54.1	104.8 ± 64.04	113.7 ± 59.7	289.2 ± 49.3	0.385	<0.001*	<0.001*	<0.001*
AFP (ng/ml)^b^	217.9 ± 215.3	-	-	-	-	-	-	-

^a^, Data presented as median (Min. – Max.), b: Data presented as mean ± SD; HCC, hepatocellular carcinoma, LC, liver cirrhosis, n, number, ALT, alanine aminotransferase, AST, aspartate aminotransferase; T-Bil, Total bilirubin; D-Bil, direct bilirubin; ALP, alkaline phosphatase; GGT, gamma glutamyl transferase; TC, Total cholesterol; TG, triglyceride; INR, international normalized ratio; Hb, hemoglobin; WBC, white blood cell; AFP, alfa fetoprotein; *P1*, comparison between HCC group and LC group; *P2*, comparison between HCC group and control group; *P3,* comparison between LC group and control group; *P4*, comparison between total cirrhosis patients and control group; *, Statistically significant at P ≤ 0.05

**Table 2:** Clinical and Radiological Characteristics of the Studied Groups

	HCC group (n = 34)	LC group (n = 36)	P
Child class, n (%)			
A	0 (0%)	2 (5.6%)	
B	12 (35.3%)	12 (33.3%)	0.655
C	22 (64.7%)	22 (61.1%)	
Child score (mean ± SD)	11.1 ± 1.9	10.4 ± 2.7	0.271
Portal hypertension, n (%)			
Yes	30 (88.2%)	30 (83.3%)	
No	4 (11.8%)	6 (16.7%)	0.736
PV thrombosis, n (%)			
Yes	12 (35.3%)	2 (5.6%)	
No	22 (64.7%)	34 (94.4%)	0.002*
Splenomegaly, n (%)			
Yes	22 (64.7%)	34 (94.4%)	
No	12 (35.3%)	2 (5.6%)	0.002*
Ascites grade, n (%)			
None	2 (5.9%)	6 (16.7%)	
Mild	6 (17.6%)	8 (22.2%)	0.321
Moderate/Severe	26 (76.5%)	22 (61.1%)	
APRI, n (%)			
F 0	0 (0%)	6 (16.7%)	
F 1	2 (5.9%)	2 (5.6%)	
F 2	2 (5.9%)	2 (5.6%)	0.102
F 3	8 (23.5%)	10 (27.8%)	
F 4	22 (64.7%)	16 (44.4%)	

HCC, hepatocellular carcinoma; LC, liver cirrhosis; n, number; PV, portal vein; APRI, AST to Platelet ratio index; P, comparison between HCC group and LC group; *, Statistically significant at P ≤ 0.05

**Table 3:** Clinical and Radiological Data in HCC Group

HCC group (n = 34)	n (%)
BCLC stage of HCC	
Early	8 (23.5%)
Intermediate	2 (5.9%)
Advanced	2 (5.9%)
End stage	22 (64.7%)
Number of FL	
Single	14 (41.2%)
Two/three	8 (23.5%)
More than three	12 (35.3%)
Size of largest FL	
Mean ± SD.	5.3 ± 2.4
Median (Min. – Max.)	5 (2.2 – 11)
Liver lobes involved	18 (52.9%)
One lobe	16 (47.1%)
Both lobes	
LN	8 (23.5%)
Extra hepatic spread	2 (5.9)

HCC, hepatocellular carcinoma; n, number; BCLC, Barcelona Clinic Liver Cancer; FL, focal lesion; LN, lymph node.

**Table 4:** Genotypes and Allele Frequency of *TGF-β1* (Arg25Pro) & *PNPLA3 *(I148M) in Total Cirrhosis Patients and Controls

	Total cirrhosis patients	Control group	P	OR (95% C.I)
	(n = 70)	(n = 20)		
*TGF-β1 *(Arg25Pro)				
GG	12/58	4/16	0.768	0.828 (0.235 – 2.917)
GC	56/14	14/6	0.343	1.7143 (0.559 –5.262)
CC	2/68	2/18	0.172	0.265 (0.035 – 2.011)
Allele frequency				
G	80/60	22/18	0.809	1.091 (0.538 – 2.213)
C	60/80	18/22	0.809	0.917 (0.452 – 1.859)
*PNPLA3* (I148M)				
CC	28/42	15/5	0.006*	0.222 (0.073 – 0.681)
(CG + GG) vs CC	42/28	5/15	0.006*	4.500 (1.470 – 13.784)
CG	30/40	5/15	0.149	2.250 (0.736 – 6.878)
GG	12/58	0/20	0.047*	–
Allele frequency				
C	86/54	35/5	0.002*	0.228 (0.084 – 0.617)
G	54/86	5/35	0.002*	4.395 (1.622 – 11.911)

OR, Odds ratio, CI, Confidence interval; *, Statistically significant at P ≤ 0.05; C, allele is the reference allele in both TGF-β1 (Arg25Pro) and PNPLA3 (I148M)

**Table 5:** Genotypes and Allele Frequency of *TGF-β1* (Arg25Pro) & *PNPLA3* (I148M) in HCC Group and LC Group

	HCC group	LC group	P	OR (95% C.I)
	(n = 34)	(n = 36)		
*TGF-β1* (Arg25Pro)				
GG	10 (29.4%)	2 (5.6%)	0.008*	7.083 (1.422 – 35.282)
GC	24 (70.6%)	32 (88.9%)	0.056	0.300 (0.084 – 1.073)
CC	0 (0%)	2 (5.6%)	0.163	–
Allele frequency				
G	44 (64.7%)	36 (50%)	0.079	1.833 (0.930 – 3.615)
C	24 (35.3%)	36 (50%)	0.079	0.546 (0.277 – 1.075)
*PNPLA3* (I148M)				
CC	14 (41.2%)	14 (38.9%)	0.845	1.100 (0.423 – 2.864)
CG	16 (47.1%)	14 (38.9%)	0.49	1.397 (0.540 – 3.612)
GG	4 (11.8%)	8 (22.2%)	0.246	0.467 (0.126 – 1.723)
Allele frequency				
C	44 (64.7%)	42 (58.3%)	0.439	1.310 (0.661 – 2.593)
G	24 (35.3%)	30 (41.7%)	0.439	0.764 (0.386 – 1.512)

HCC, hepatocellular carcinoma; LC, liver cirrhosis; OR, Odds ratio; CI, Confidence interval; *, Statistically significant at P≤ 0.05; C, allele is the reference allele in both TGF-β1 (Arg25Pro) and PNPLA3 (I148M)

**Table 6:** The relation between *TGF-β1* (Arg25Pro) & *PNPLA3 *(I148M) Variants and the Different Studied Parameters

	*PNPLA3*			*TGF- β1*		
	C (n =86)	G (n = 54)	P	GG	GC+CC	P
				(n = 12)	(n =58)	
Gender, n (%)				3		
Male	44 (51.2%)	36 (66.7%)	0.071	8 (66.7%)	32 (55.2%)	0.464
Female	42 (48.8%)	18 (33.3%)		4 (33.3%)	26 (44.8%)	
Age ^b^	60.4 ± 7.3	58 ± 5.8	0.036*	56.8 ± 6.1	60 ± 6.9	0.141
Child class, n (%)						
A	2 (2.3%)	2 (3.7%)	0.894	0 (0%)	2 (3.4%)	1
B	30 (34.9%)	18 (33.3%)		4 (33.3%)	20 (34.5%)	
C	54 (62.8%)	34 (63%)		8 (66.7%)	36 (62.1%)	
Child score ^b^	10.7 ± 2.3	10.7 ± 2.4	0.993	11 ± 1.9	10.7 ± 2.4	0.679
Portal hypertension, n (%)						
Yes	72 (83.7%)	48(88.9%)	0.395	10 (83.3%)	50(86.2%)	0.678
No	14 (16.3%)	6(11.1%)		2 (16.7%)	8(13.8%)	
Ascites grade, n (%)						
None	8 (9.3%)	8 (14.8%)	0.599	2 (16.7%)	6(10.3%)	0.113
Mild	18 (20.9%)	10 (18.5%)		0 (0%)	14(24.1%)	
Moderate/Severe	60 (69.8%)	36 (66.7%)		10 (83.3%)	38(65.5%)	
ALT (U/L)^ a^	36 (12 – 291)	35 (12 – 134)	0.244	57.5 (23 – 291)	35 (12 – 234)	0.057
AST (U/L)^ a^	65 (21 – 381)	67 (21 – 257)	0.824	129.5 (34 – 381)	67 (21 – 312)	0.17
Albumin (g/dl) ^b^	2.3 ± 0.4	2.3 ± 0.4	0.79	1.9 ± 0.4	2.3 ± 0.3	0.001*
T-Bil (mg/dl)^ a^	2.5 (0.5 – 28.1)	3.5(0.5 – 23.2)	0.329	5.5 (1.4 – 16.7)	2.6 (0.5 – 28.1)	0.105
D-Bil (mg/dl)^ a^	1.6 (0.1 – 22.6)	1.7 (0.2 – 16.4)	0.4	4.5 (0.7 – 12.7)	1.6 (0.1 – 22.6)	0.049*
ALP (U/L)^ a^	89 (33 – 250)	112 (42 – 275)	0.007*	150.5 (65 – 182)	89 (33 – 275)	0.212
GGT (U/L)^ a^	35 (14 – 164)	56 (19 – 176)	0.015*	75.5 (20 – 164)	42 (14 – 176)	0.236
TC (mg/dl) ^b^	156 ± 49.5	162.2 ± 63.6	0.545	123.7 ± 31.6	165.6 ± 56.7	0.016*
TG (mg/dl) ^b^	118.7 ± 31	131 ± 45.7	0.085	122 ± 31.1	123.7 ± 39.2	0.889
INR ^b^	1.6 ± 0.4	1.5 ± 0.3	0.111	1.7 ± 0.3	1.6 ± 0.4	0.317
Hb (g/dl) ^b^	10.1 ± 1.7	10.4 ± 1.1	0.176	10.5 ± 1.6	10.2 ± 1.5	0.553
WBC count (x10^3^/μL) ^a^	9.2 (2.6 – 30.4)	8.3 (3.8 – 30.4)	0.084	11.3 (7.9 – 28.2)	8.3 (2.6 – 30.4)	0.004*
Platelets count (x10^3^/μL) ^b^	106.7 ± 57.1	124.7 ± 61.9	0.082	94.8 ± 36.9	117.6 ± 62.9	0.104
APRI, n (%)						
Early(F0, F1, F2)	14 (16.3%)	14(25.9%)	0.165	4(33.3%)	10(17.2%)	0.24
Late (F3, F4)	72 (83.7%)	40(74.1%)		8(66.7%)	48(82.8%)	

^a^, Data presented as median (Min. – Max.); ^b^, Data presented as mean ± SD; ALT, alanine aminotransferase; AST, aspartate aminotransferase; T-Bil, Total bilirubin; D-Bil, direct bilirubin; ALP, alkaline phosphatase; GGT, gamma glutamyl transferase; BUN, blood urea nitrogen; INR, international normalized ratio; Hb, hemoglobin; WBC, white blood cell; APRI, AST to Platelet ratio index; *, Statistically significant at P ≤ 0.05

## Discussion

Chronic HCV infection is considered the leading causes of end-stage liver disease, hepatocellular carcinoma, and consequently liver transplantation worldwide (Fernández-Rodríguez et al., 2013). Hepatic fibrosis is recognized as a common hallmark of chronic liver disease (Krawczyk et al., 2011), and HCC risk increases with the severity of inflammation and fibrosis (Migita et al., 2005). Because the progress of fibrosis differs markedly between individuals with similar risk profiles, there is increasing evidence that host genetic factors may play an important role in cirrhosis development in chronic HCV infection (Fernández-Rodríguez et al., 2013). To give an insight on the molecular factors underlying the evolution of liver cirrhosis to HCC, we evaluated the association between the genetic variations in two pro-fibrogenic genes; TGF-β1 and PNPLA3 and the progression of chronic HCV infection, liver cirrhosis development, and HCC risk among patients with HCV infection (Lee and Friedman, 2011; Dongiovanni et al., 2013).

TGF-β1 is a pleiotropic cytokine that has been linked to fibrosis and neoplasm of the liver. It was reported that this cytokine promotes the transcription, synthesis and secretion of numerous extracellular matrix proteins including collagens, fibronectin, and proteoglycans, in addition to its fibrogenic action leading to hepatic stellate cells (HSCs) trans differentiation into myofibroblasts which is considered a crucial biological step in hepatic fibrogenesis (Dewidar et al., 2019). 

It also plays a role in the regulatory immune responses to viral infections by increasing the number and activation of T regulatory lymphocytes, as well as the recruitment of these cells to the infected liver, which leads to prolonged chronic, asymptomatic, and occult forms of infection (Karimi-Googheri et al., 2014). It was reported that the transition of G to C in the locus of codon 25 (G>C) may impact the production of TGF-β1 and it was correlated with the reduced levels of TGF-β1 in vitro. However, the subjects with codon 25 G allele which was associated with high TGF-β1 production displayed over suppression in the human immune response. This mechanism may elucidate the correlation of the polymorphism of Arg25Pro (G>C) and chronic HCV infection (Guo et al., 2019). 

Although several epidemiological studies have examined the association of TGF-β1 Arg25Pro (G>C) variant with the chronic HCV infection susceptibility, and fibrosis progression, controversial results still exist (Gewaltig et al., 2002; Pereira et al., 2008; Guo et al., 2019). In the current study we did not find significant difference in genotyping distribution or allele frequency of TGF-β1 (Arg25Pro) variant when comparing total cirrhosis patients with controls. Also, no association was found between GG genotype of TGF-β1 (Arg25Pro) and APRI score (P= 0.240). In accordance with our results, (Hosseini Razavi et al., 2014) observed non-statistically significant differences in terms of genotype distribution or allele frequency of this polymorphism between patients with chronic hepatitis and healthy controls in Iranian population. In another study, (Romani et al., 2011) reported the same result. Also, in consistent with our finding, a meta-analysis which was conducted on eight eligible case control studies, including a total of 910 cases and 632 controls demonstrated that there is not any association between TGF-β1 (Arg25Pro) variant and the susceptibility to chronic HCV infection (Hu et al., 2011). Another meta-analysis conducted by (Gou et al., 2019) on 2718 chronic HCV infection cases confirmed the same finding for this TGF-β1 variant.

In the present study, GG genotype distribution of TGF-β1 (Arg25Pro) was significantly higher in HCC group than HCV group (7.083 (1.422 – 35.282)) (Table 5). It was described that TGF- β1 act as tumor suppressor in early carcinogenesis by inducing apoptosis or inhibiting cell growth, while promoting tumorigenicity and metastasis through the local and systemic immunosuppression effect and supporting tumor progression in advanced stages of cancer (Seoane et al., 2017; Xia et al., 2018; Hadj-Ahmed et al., 2019).

Mouse experiments showed that altered TGF-β1 was associated with the latent TGF-β1 binding proteins that can cause inflammation and tumors (Guan et al., 2009). Also, the persistence of chronic inflammation, as observed in chronic viral hepatitis, plays a major role in determining the shift in the TGF-β1 signalling pathway from tumour suppression to fibrogenesis which accelerate liver fibrosis and increase the risk for HCC. Therefore, the variants in TGF-β1 will alter the level of protein expression, which may affect the susceptibility to tumor development including HCC (Radwan et al., 2012). TGF-β1 signaling pathway was reported to exert potent pro-oncogenic properties, and disruption of the pathway is frequently observed in a wide spectrum of chronic liver diseases and fibrogenesis, as well as liver cancer development. Moreover, alterations of TGF-β1 signaling are associated with unfavorable tumor biology (Marquardt et al., 2018).

Many variants of TGF-β1 including +869 C>T and –509 C>T were previously demonstrated to be associated with susceptibility to HCC (Guo et al., 2013; Lu et al., 2016; Toshikuni et al., 2016), however the present work is considered the first to identify Arg25Pro variant to be related to HCC risk in Egyptian population. On the reverse of our result, the study conducted by (Falleti et al., 2008) didn’t find a relation between TGF-β1 (Arg25Pro) genotypes or allele frequency and the occurrence of HCC in Italian population.

In this study, GG genotype and G allele distributions of PNPLA3 (I148M) variant were significantly higher in total cirrhosis patients than controls (P= 0.047), (OR: 4.395, CI 95%: 1.622–11.911)(Table 4) respectively. This result was in agreement with (Ali et al., 2016) who reported that the patients have PNPLA3 CG/GG genotype were at nearly two-fold increased odds of fibrosis progression giving a higher risk for cirrhosis and secondary complications. Also Manchiero et al., (2017) demonstrated an association between the genotype GG, hepatic steatosis, and progressive fibrosis in Brazilian patients. Similarly, (Huang et al., 2015) reported the same results in Taiwanese population. Moreover, (Fan et al., 2016) determined in their meta-analysis that PNPLA3 (I148M) GG was associated with the risk of both advanced liver fibrosis and steatosis among Caucasians but not Asian populations, and they confirmed the relatedness of this polymorphism and liver disease susceptibility in HCV infection patients.

The biological role of PNPLA3 is debated, but it likely facilitates accumulation of fat in hepatocytes, potentially by interfering with the export of lipoprotein and favoring lipogenic activity over lipase activity (Ali et al., 2016). Using recombinant PNPLA3 assays in vitro confirmed that the wild-type enzyme has the ability to hydrolyze emulsified TG, and the substitution of I148M decreases this activity, also the expression of PNPLA3 (I148M) GG in hepatocytes culture and in the mice livers augmented cellular TG contents (Manchiero et al., 2017). Another study suggested that the I148M variant’s effect on fibrosis was independent of its effect on hepatic steatosis and inflammation, implying that it has an effect on the quantity and quality of hepatic lipids and on the biology of non-parenchymal liver cells as well on hepatocytes, which directly promote fibrogenesis. As a result, PNPLA3 is considered as a central player in the development of liver disease. (Pirazzi et al., 2014) demonstrated that PNPLA3 is highly expressed in human HSCs indicating a possible correlation between HSCs, PNPLA3, and retinoid metabolism in determining vulnerability to hepatic fibrosis. Another report showed that human HSCs having PNPLA3 (I148M) GG variant expressed more inflammatory cytokines and chemokines such as granulocyte-macrophage colony-stimulating factor, TGF-β, and chemokine (C-X-C motif) ligand 8. Overexpression of the PNPLA3 (I148M) GG variant augments the HSCs proliferation and chemotaxis (Bruschi et al., 2017). Further signaling analysis showed that PNPLA3 (I148M) GG HSCs have high activity of c-Jun N-terminal kinase (JNK), as a result, the main HSCs quiescence regulator; peroxisome proliferator-activated receptor gamma is inhibited, while activator protein 1, a proinflammatory transcription factor, is activated (Dong, 2019). These dysregulations taken together lead to the fibrogenic phenotype in the PNPLA3 (I148M) GG HSCs.

In this work, we also evaluated the association between PNPLA3 (I148M) variant and HCC. We didn’t demonstrate a significant difference in genotyping or allele frequency of PNPLA3 (I148M) variant when comparing HCC group with LC group.

In consistence with our result, (Yen et al., 2018) affirmed that there is no influence of the PNPLA3 (I148M) GG genotype on the occurrence of HCC in cirrhotic patients. Hai et al., (2017) reported the same results in Japanese patients. Also, Ali et al., (2016) confirmed the same result in HCV-infected patients, suggesting this association may be isolated to patients with non-viral liver disease. On the other hand, other studies reported an increased risk of HCC development in patients carrying the G allele with alcoholic cirrhosis (Liu et al., 2014; Seko et al., 2017; Grimaudo et al., 2020), and nonalcoholic fatty liver disease (NAFLD).

While PNPLA3 (I148M) variant has been convincingly linked to liver carcinogenesis in alcoholic liver disease and in steatohepatitis, the relation in viral hepatitis has been subjected to controversial results. This discrepancy may be due to the diverse ethnicities of the studied populations, as well as variable genetic contributors, other polymorphisms, or environmental factors which might explain the observed differences among ethnic groups.

In conclusion, our findings suggest that TGF-β1 (Arg25Pro) variant may be one of the genetic factors affecting hepatic carcinogenesis in patients with HCV infection, and might play a role in HCC susceptibility but it is not associated with the risk of progression to chronic HCV infection and consequently liver cirrhosis in Egyptian population. PNPLA3 (I148M) variant is likely to hasten the progression to liver cirrhosis, but they appear to have no role in predicting the occurrence of HCC. Identification of high-risk patients could help identify a subgroup of patients in which liver function should be closely monitored, treatment of HCV could be considered early, and attempts to track HCC could be targeted.

 Our study had certain limitations that need to be acknowledged. These include the small sample size used in the study and the limited number of SNPs analyzed which would provide a broader and clearer prospective understanding of the insight on the molecular factors underlying the evolution of liver cirrhosis to HCC.

## Author Contribution Statement

Noha Said Kandil: Selection of the research idea and research design, participation in the writing and reference gathering of the paper, participation in the performance of molecular laboratory tests, data interpretation, plagiarism checking and responsible for the international publishing process.

Azhar Mohamed Nomair: Participation in the writing and reference gathering of the paper, participation in the performance of molecular laboratory tests, results and data interpretation, statistical analysis, plagiarism checking and paper revision.

Lamia Said Kandil: Participation in the writing and literature review and reference gathering of the paper, participation in the performance of molecular laboratory tests, english editing and paraphrasing and paper revision.

Hanan Mohamed Nomeir: Participation in the writing and reference gathering of the paper, participation in the performance of molecular laboratory tests, data interpretation and paper revision.
